# Case report: Immunological characteristics of *de novo* ulcerative colitis in a child post COVID-19

**DOI:** 10.3389/fimmu.2023.1107808

**Published:** 2023-02-16

**Authors:** Atsushi Morita, Kazuo Imagawa, Manabu Tagawa, Noriaki Sakamoto, Hidetoshi Takada

**Affiliations:** ^1^ Department of Pediatrics, University of Tsukuba Hospital, Tsukuba, Japan; ^2^ Department of Child Health, Faculty of Medicine, University of Tsukuba, Tsukuba, Japan; ^3^ Department of Diagnostic Pathology, Faculty of Medicine, University of Tsukuba, Tsukuba, Japan

**Keywords:** cytokine - immunological terms, gut barrier, inflammatory bowel disease, lipopolysaccharide-binding protein (LBP), multisystem inflammatory syndrome in children (MIS-C), SARS-CoV-2 spike protein, T cell receptor (TCR), zonulin

## Abstract

The pathological mechanisms of *de novo* inflammatory bowel disease (IBD) following SARS-CoV-2 infection are unknown. However, cases of coexisting IBD and multisystem inflammatory syndrome in children (MIS-C), which occurs 2–6 weeks after SARS-CoV-2 infection, have been reported, suggesting a shared underlying dysfunction of immune responses. Herein, we conducted the immunological analyses of a Japanese patient with *de novo* ulcerative colitis following SARS-CoV-2 infection based on the pathological hypothesis of MIS-C. Her serum level of lipopolysaccharide-binding protein, a microbial translocation marker, was elevated with T cell activation and skewed T cell receptor repertoire. The dynamics of activated CD8^+^ T cells, including T cells expressing the gut-homing marker α4β7, and serum anti-SARS-CoV-2 spike IgG antibody titer reflected her clinical symptoms. These findings suggest that SARS-CoV-2 infection may trigger the *de novo* occurrence of ulcerative colitis by impairing intestinal barrier function, T cell activation with a skewed T cell receptor repertoire, and increasing levels of anti-SARS-CoV-2 spike IgG antibodies. Further research is needed to clarify the association between the functional role of the SARS-CoV-2 spike protein as a superantigen and ulcerative colitis.

## 1 Introduction

Inflammatory bowel diseases (IBDs), such as Crohn’s disease and ulcerative colitis, are characterized by chronic intestinal inflammation. The underlying pathophysiology of IBD is induced by impaired intestinal barrier function due to genetic or environmental triggers, and IBD develop in genetically susceptible hosts because of dysregulated immune responses (1, 2). The altered balance of effector and regulatory T cells can lead to development of inflammatory conditions accompanied by expansion of specific pathogenic T cell clones (3).

IBD may be triggered by infections in some patients (2). After the coronavirus disease-2019 (COVID-19) pandemic, several reports have associated the *de novo* occurrence of IBD in both adults and children with severe acute respiratory syndrome coronavirus 2 (SARS-CoV-2) infection, and most of those IBDs developed several weeks after SARS-CoV-2 infection (4–8). Moreover, two cases of IBD occurring after the onset of the multisystem inflammatory syndrome in children (MIS-C), which occurs 2–6 weeks after SARS-CoV-2 infection, have been reported (9, 10). Although the pathological mechanism shared between IBD and SARS-CoV-2 infection is unknown, coexisting IBD and MIS-C is reminiscent of a shared underlying dysfunction of immune responses. In MIS-C, SARS-CoV-2 persistence in the gastrointestinal tract can increase gut permeability, allowing the superantigen motif of the SARS-CoV-2 spike protein, which resembles to Staphylococcal enterotoxin B (11), to travel across mucosal barriers and into the bloodstream, triggering widespread T cell activation (12). This pathophysiological condition of MIS-C, which is induced by impaired intestinal barrier function, may have some similarity with that of IBD.

In this study, we report the case of a pediatric patient with ulcerative colitis after SARS-CoV-2 infection and conducted longitudinal analyses of gut permeability markers as well as peripheral immunophenotyping based on the pathological hypothesis of MIS-C. This case provides insights into the role of the SARS-CoV-2 superantigen as well as the pathophysiology of ulcerative colitis post-SARS-CoV-2 infection and supports the hypothesis that SARS-CoV-2 infection triggers the *de novo* occurrence of ulcerative colitis.

## 2 Patient and methods

### 2.1 The patient

A 13-year-old Japanese girl was admitted to the University of Tsukuba Hospital. Immunological analyses were conducted in the context of a research project (R01-249) approved by the hospital ethics committee. Written informed consent was obtained from both the patient and her parents. The month of ulcerative colitis onset, when gastrointestinal symptoms first appeared, was defined as month 0. Each clinical sample was collected from the patient in month 2, 2.5, 3.5, 5, and 6, and the sera were stored at -80 °C. Month 2 was before the treatment of ulcerative colitis began and 13 days after the first vaccination with BNT162b2. Month 2.5 was 1 day after the second vaccination with BNT162b2.

### 2.2 Analysis of serum gut permeability markers

Serum zonulin and lipopolysaccharide-binding protein (LBP) levels were measured using a zonulin (serum) ELISA kit (Immundiagnostik, Bensheim, Germany) and an LBP ELISA kit (Hycult Biotech, Wayne, PA, USA), respectively according to the manufacturer’s instructions. Seven cases of ulcerative colitis in remission and six cases of Crohn’s disease in remission were used as negative controls, and one case of MIS-C was used as the positive control (see reference (13) for details on the clinical course of MIS-C).

### 2.3 Immunophenotyping of T cells

Immune cells from whole peripheral blood were phenotyped using three separate flow cytometry panels (**Supplementary Table 1**), which were created based on the Human Immunology Project (14). If the white blood cells count exceeding 10×10^3^ cells/µL, the whole blood was diluted with phosphate-buffered saline to achieve counts in the range of 3–10×10^3^ cells/µL. Subsequently, 100 µL of whole blood and each antibody reagent were added to a Coulter tube and incubated for 20 minutes. Then, TQ-Prep automatically added lysis buffer, stabilizer and fixative to the tubes using the Immunoprep Reagent System (Beckman Coulter Inc., Brea, CA, USA). For data acquisition, a BD LSRFortessa flow cytometer (Becton Dickinson, Franklin Lakes, NJ, USA) was used and data were analyzed using FlowJo (10.6.2; Treestar). The gating strategies are outlined in **Supplementary Figures 1**-**3** using a patient sample from month 2. Each T cell subset count was obtained by multiplying the subset percentage by the anchor marker percentage of the total CD45 lymphocyte population multiplied by the absolute lymphocyte count.

### 2.4 Analysis of T-cell receptor repertoire

Phenotypic analysis of whole blood for the T cell receptor (TCR) Vβ repertoire was performed on month 2 using the IOTest Beta Mark kit (Beckman Coulter) containing 24 monoclonal antibodies to identifying approximately 70% of the T cell repertoire. Whole blood was stained with surface markers in eight sample tubes (**Supplementary Table 2**). All samples were acquired on a BD LSRFortessa flow cytometer (Becton Dickinson) and analyzed using FlowJo (10.6.2; Treestar). Lymphocytes were first gated according to the FSC/SSC parameter, followed by the selection of CD3^+^CD4^+^CD45RO^+^, CD3^+^CD8^+^CD45RO^+^, CD3^+^CD4^+^CD38^+^HLA-DR^+^, and CD3^+^CD8^+^CD38^+^HLA-DR^+^ positive cells.

### 2.5 Analysis of serum cytokines and chemokines

The concentrations of interleukin-1β (IL-1β), IL-2, IL-4, IL-5, IL-6, IL-8, IL-10, IL-12/IL-23p40, IL-13, IL-17A, IL-21, tumor necrosis factor (TNF), interferon-γ (IFN-γ), monocyte chemotactic protein 1 (MCP-1), CXCL9 known as monokine-induced by interferon-γ (MIG), CXCL10 known as interferon-γ-induced protein-10 (IP-10), and eotaxin were measured in serum samples using a BD LSRFortessa flow cytometer (Becton Dickinson) with BD Cytometric Bead Array Flex Sets and a Human Th1/Th2/Th17 CBA Kit (Becton Dickinson), according to the manufacturer’s instructions.

### 2.6 Analysis of anti-SARS-CoV-2 spike IgG antibody

Serum anti-SARS-CoV-2 spike IgG antibody levels were measured using a human SARS-CoV-2 spike (Trimer) IgG ELISA kit (Thermo Fisher Scientific, Waltham, MA, USA) according to the manufacturer’s instructions.

### 2.7 Detection of fecal Staphylococcal enterotoxin and fecal SARS-CoV-2 as super antigen motifs

Both small and large intestinal fluid were collected from a patient during ileocolonoscopy at month 2. Each fluid sample was centrifuged for 30 min at 5,000×*g*, and the supernatants were stored at -80 °C. For the polymerase chain reaction (PCR) analysis of SARS-CoV-2, the supernatants were further centrifuged for 5 min at 12,000×*g*, and stored at -80 °C, and RNA was extracted using the QIAamp Viral RNA Mini kit (QIAGEN, Hilden, Germany) according to the manufacturer’s instructions. Staphylococcal enterotoxin levels were measured using RIDASCREEN SET A,B,C,D,E (R-Biopharm AG, Darmstadt, Germany) according to the manufacturer’s instructions. PCR for SARS-CoV-2 was performed in accordance with a previously reported protocol (15).

## 3 Results

### 3.1 Clinical course

**Figure 1:**
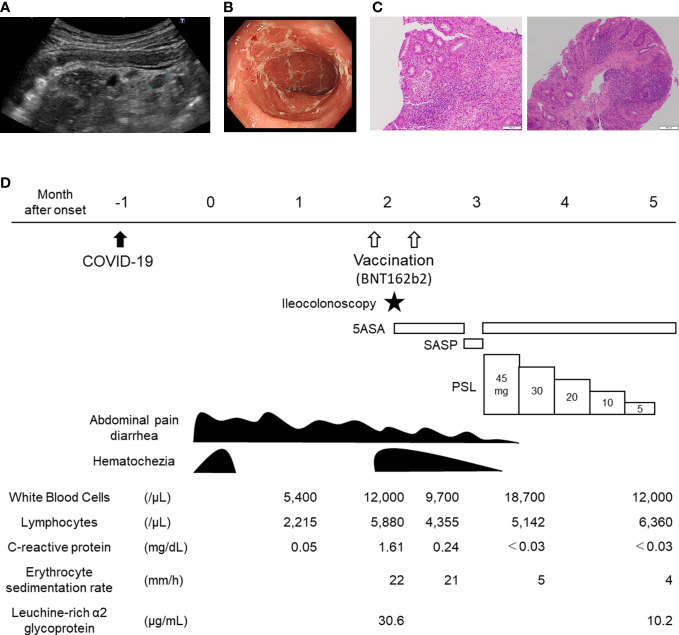
Clinical course of the 13-year-old patient with *de novo* ulcerative colitis post COVID-19. **(A)** Abdominal ultrasound depicting sigmoid colon. Wall thickening and loss of Haustra with mesenteric lymphadenopathy (dotted line) are shown. **(B)** Endoscopic image depicting sigmoid colon. Complete obliteration of vascular pattern with erosions are shown. **(C)** histology of ascending colon (left) and rectum (right): A crypt abscess in the ascending colon and crypt distortion in the rectum are shown. **(D)** Clinical course of the patient. COVID-19, coronavirus disease 2019; 5ASA, 5-aminosalicylate; PSL, Prednisolone; SASP, salazosulfapyridine.

A previously healthy 13-year-old Japanese girl was transferred to our hospital because of hematochezia. The patient’s initial presentation included abdominal pain, diarrhea, and hematochezia, which occurred one month after acquiring self-limiting COVID-19. During her COVID-19, the patient had fever, cough, and dysgeusia, but no gastrointestinal symptoms. The primary care center prescribed the patient an antiflatulent, and her hematochezia subsided; however, abdominal pain and diarrhea persisted. Two months after the onset of gastrointestinal symptoms, the patient was vaccinated with BNT162b2, and hematochezia recurred. The patient was then transferred to our hospital. On arrival, the patient tested negative for SARS-CoV-2 by PCR analysis of a nasopharyngeal swab. Clinical examination yielded normal results and the patient did not exhibit weight loss. However, abdominal ultrasound revealed wall thickening and loss of haustra in the entire colon with mesenteric lymphadenopathy (**Figure 1A**). Laboratory investigations indicated chronic inflammation with intestinal inflammation (erythrocyte sedimentation rate, 22 mm/h; white blood cells, 12,000/µL; c-reactive protein, 1.61 mg/dL; immunoglobulin G, 2250 mg/dL; serum amyloid A, 36.3 µg/mL; serum proteinase 3 antineutrophil cytoplasmic antibodies, 32.3 U/mL; hemoglobin 11.4 g/dL; serum iron, 8 U/mL; ferritin, 11.0 µg/mL; leucine-rich alpha-2 glycoprotein, 30.6 µg/mL; fecal calprotectin, 6,110 mg/kg). Serology and PCR testing ruled out Epstein-Barr virus and cytomegalovirus infections. Blood and fecal cultures were negative. Ileocolonoscopy revealed complete obliteration of the vascular pattern of the colon with erosions and some luminal bleeding (**Figure 1B**). Histological assessment of the biopsy specimen revealed a crypt abscess and crypt distortion (**Figure 1C**). Based on these findings, the patient was diagnosed with pancolitis type of ulcerative colitis. The pediatric ulcerative colitis activity index (16), ulcerative colitis endoscopic index of severity (17), and Geboes histopathology score (18) at the time of diagnosis were 65 (severe), 6 (moderate), and 5.4 (Grade 5), respectively. The patient was administered 5-aminosalicylate therapy. However, the patient did not reach remission. Although there was no obvious exacerbation of clinical symptoms after second vaccination of BNT162b2, hematochezia persisted and switching to salazosulfapyridine was ineffective. The patient was subsequently administered steroid therapy, which was effective and she achieved remission. (**Figure 1D**).

### 3.2 Changes in gut permeability markers

**Figure 2:**
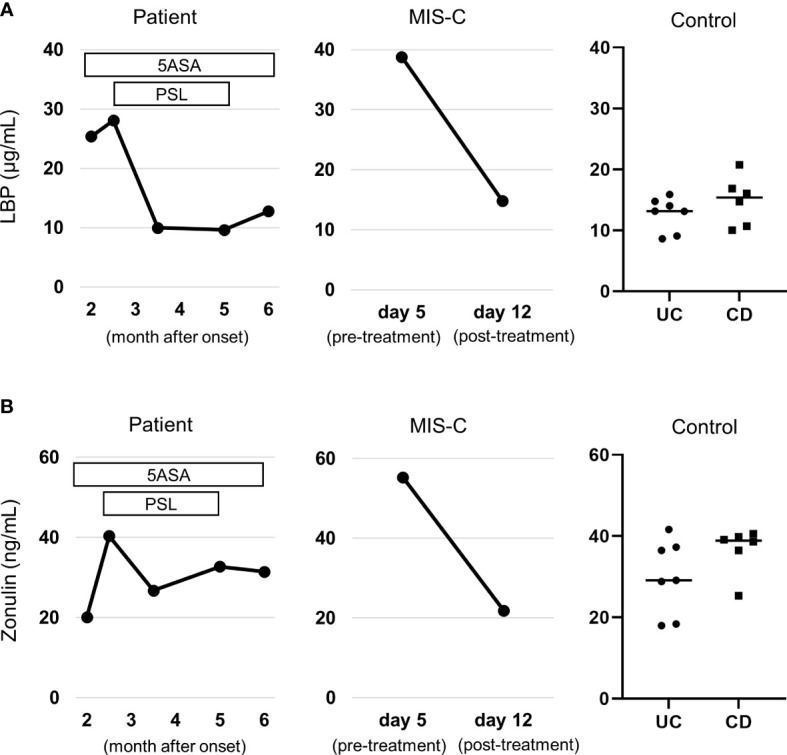
Serum gut permeability markers. **(A)** Changes in serum zonulin. **(B)** Changes in serum lipopolysaccharide binding protein (LBP). Levels of both markers were compared to those patients with MIS-C as positive control (see reference (13) for details on the clinical course) and patients with inflammatory bowel disease in remission as negative control. Note that the patient did not have elevated zonulin through the clinical course, but had elevated LBP, which was normalized by steroid therapy. They were measured by a zonulin (serum) ELISA kit (Immundiagnostik, Bensheim, Germany) and an LBP ELISA kit (Hycult Biotech, Wayne, PA, USA), respectively. 5ASA, 5-aminosalicylate; PSL, Prednisolone; UC, ulcerative colitis; CD, Crohn’s disease; MIS-C, multisystem inflammatory syndrome in children.

First, we confirmed that our previously reported MIS-C patients (13) had elevated levels of both serum zonulin and LBP during the pre-treatment acute phase, consistent with a previous report (12). Compared to the MIS-C patient as a positive control and the IBD patients in remission as negative control, the patient had elevated LBP during the active period (**Figure 2A**), and LBP did not decrease 5-aminosalicylate therapy, but decreased steroid therapy. However, the patient did not show elevated zonulin levels throughout the course (**Figure 2B**).

### 3.3 Histological immunophenotyping and changes in peripheral T-cell subsets

**Figure 3:**
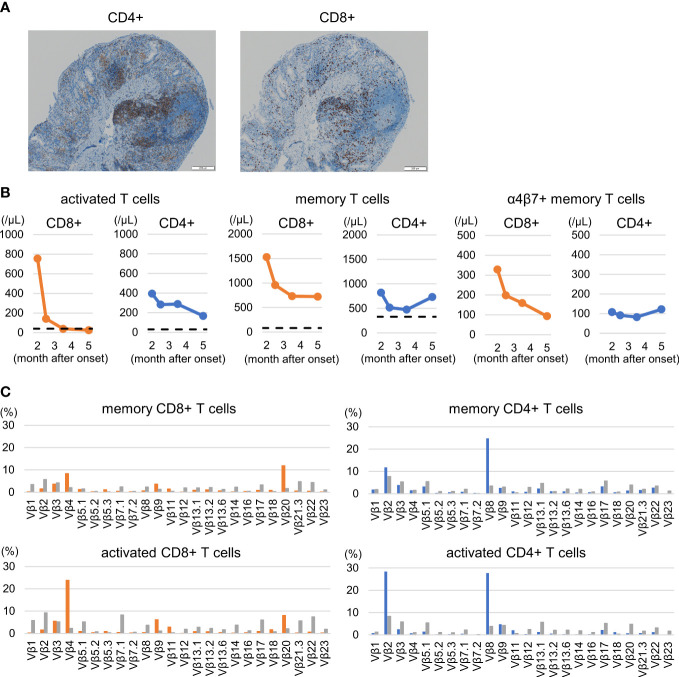
Colorectal histological and peripheral immunophenotyping. **(A)** Rectal histology: Moderate to severe lymphocyte infiltration and equally composed of CD8^+^ and CD4^+^ lymphocytes are shown. **(B)** Changes in T cell subsets: activated T cells (HLA-DR^+^ CD38^+^), memory T cells (CD45RO^+^), and α4β7^+^ memory T cells. Increases in activated and memory T cells, including α4β7-expressing cells, particularly CD8+ T cells, are shown. The dashed lines indicate the median value of healthy children around the same age as the patient according to reference values (19). **(C)** Changes in T cell receptor repertoire during the active pre-treatment period. The expansion of Vβ 4 and Vβ 20 in memory CD8^+^ T cells and of Vβ 2 and Vβ 8 in memory CD4^+^ T cells were observed. A similar trend was observed in activated T cells. The gray bar indicates the control value of a healthy 11-year-old boy. See supplementary materials of panel design and gating strategy for peripheral T-cell subsets and T-cell receptor repertories.

Histological assessment of the biopsy specimen revealed moderate to severe lymphocyte infiltration and equally composed of CD8^+^ and CD4^+^ lymphocytes. (**Figure 3A**). In peripheral immunophenotyping, we observed an increase in activated T cells (HLA-DR^+^ CD38^+^) and memory T cells (CD45RO^+^) during active phase. Notably, changes in the activation of CD8^+^ T cells were remarkable, and the decrease was observed to be correlated with improvement of clinical symptoms. Moreover, CD8^+^ memory T cells expressing α4β7, which is an intestinal homing molecule and is a target of vedolizumab, also reflected clinical symptoms (**Figure 3B**). No remarkable changes were observed in Th1, Th2, Th17 cells, follicular T cells, or regulatory T cells (data not shown).

### 3.4 T-cell receptor repertories

Increases of Vβ 4 and Vβ 20 in memory CD8^+^ T cells and of Vβ 2 and Vβ 8 in memory CD4^+^ T cells were observed on the pretreatment active period (**Figure 3C** upper). A similar trend was observed for CD38^+^ HLA-DR^+^ CD4^+^ or CD8^+^ T cells (**Figure 3C** lower). Vβ 21.3 expansion, known as a hallmark of MIS-C (13, 20), was not observed.

### 3.5 Changes in serum cytokines and chemokines

**Figure 4:**
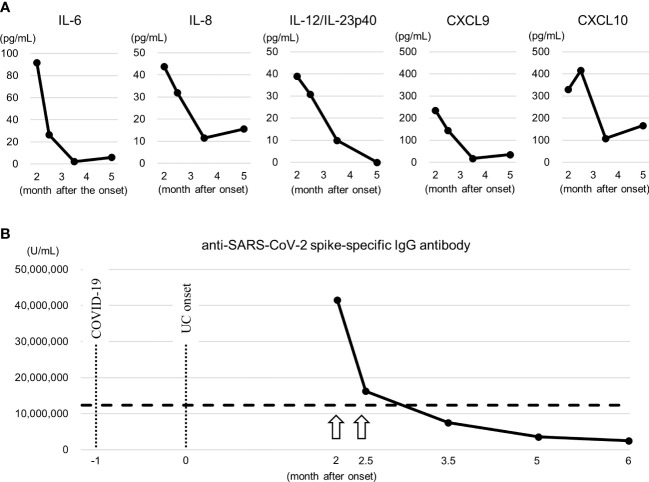
Serum cytokines and anti-SARS-CoV-2 spike IgG antibody titer. **(A)** Changes in serum cytokines and chemokines. These elevated cytokines and chemokines suggest the activation of cell-mediated, especially CD8^+^ T cells, and innate inflammatory immunity. They were measured by a Cytometric Bead Array (Becton Dickinson, Franklin Lakes, NJ, USA). **(B)** Changes in anti-SARS-CoV-2 spike IgG antibody titer. The arrow indicates the time of vaccination of BNT162b2. Dashed line represents highest value in the patient with MIS-C (13). Note that at the month 2.5, 1 day after the second vaccination, no increase in the titer exceeding the titer after the first vaccination was confirmed, and no apparent exacerbation of clinical symptoms was noted at the time. They were measured by a human SARS-CoV-2 spike (Trimer) IgG ELISA kit (Thermo Fisher Scientific, Waltham, MA, USA). COVID-19, coronavirus disease 2019; CXCL, chemokine (C-X-C motif) ligand; IL, interleukin; UC, ulcerative colitis.

The active disease period was characterized by increased serum levels of a several cytokines and chemokines, including IL-6, IL-8, IL-12/IL-23p40, CXCL9, and CXCL10 (**Figure 4A**), suggesting the activation of cell-mediated and innate inflammatory immunity. In particular, elevated IL-12/IL-23p40, CXCL9, and CXCL10 levels further support the involvement of CD8^+^ T cells (21). No remarkable changes were observed in the levels of the other cytokines and chemokines.

### 3.6 Changes in anti-SARS-CoV-2 spike IgG antibody

The anti-SARS-CoV-2 spike IgG antibody titer was significantly higher than the highest value in the patient with MIS-C (13) at month 2, 13 days after the first vaccination, and the change in the titer reflected the clinical symptoms (**Figure 4B**), similar to CD8^+^ T cells. At the month 2.5, 1 day after the second vaccination, no increase in the titer exceeding the titer after the first vaccination was confirmed, and no apparent exacerbation of clinical symptoms was noted at the time.

### 3.7 Search for superantigens

Because of the remarkable T cell activation with a skewed T cell receptor repertoire, we searched for superantigen motifs. However, neither Staphylococcal enterotoxin nor SARS-CoV-2 antigen was detected in either the small or large intestinal fluid (data not shown).

## 4 Discussion

We clarified the detailed immunological characteristics of a Japanese patient with *de novo* ulcerative colitis following SARS-CoV-2 infection. We confirmed elevated serum LBP, a microbial translocation marker, with T cell activation and skewed T cell receptor repertoire. The dynamics of activated and memory CD8^+^ T cells, including T cells expressing the gut-homing marker α4β7, and serum anti-SARS-CoV-2 spike IgG antibody titer reflected her clinical symptoms. These findings suggest that SARS-CoV-2 infection may trigger the *de novo* occurrence of ulcerative colitis by impairing barrier function in the intestinal mucosa, T cell activation with a skewed T cell receptor repertoire, and increased anti-SARS-CoV-2 spike IgG antibody titers.

We observed elevated serum LBP, but not serum zonulin, in the patient. Zonulin belongs to a family of structurally and functionally related proteins that reversibly regulate intestinal permeability by modulating intercellular tight junctions. Increased circulating zonulin levels result in increased intestinal permeability (22). However, increased LBP levels reflect the recent microbial translocation (22). Interestingly, while it has been reported that patients with ulcerative colitis have increased in LBP level (23), there are conflicting reports on zonulin level (24, 25). Together with these previous reports, the current case leads us to speculate that while zonulin may be involved in the acute impairment of intestinal barrier function, it is not involved in the chronic impairment at least two months after the onset. Rather, it is possible that T cell-mediated immune reaction play a central role in chronic impairment of barrier function. However, further investigation including comparison of fecal and serum zonulin is needed.

We also found increased T cell activation, especially that of CD8^+^ T cells, with a skewed T cell receptor repertoire in the patient, suggesting superantigen-mediated T cell activation. In addition, memory CD8^+^ T cells expressing gut-homing marker α4β7 proliferated and correlated with changes in clinical symptoms. Furthermore, histological findings showed the infiltration of CD8^+^ and CD4^+^ lymphocytes. The pathological involvement of T cells was evident from these observations. Although there are limited data concerning whether TCR repertoires are altered in inflammatory bowel disease, the clonal expansion of TCR-β-expressing T cells in pediatric patients with ulcerative colitis correlates with disease severity, suggesting their involvement in mediating intestinal inflammation (3). The current patient manifested a clinically severe ulcerative colitis and accompanied with considerably skewed T cell receptor repertoire. Unfortunately, we were unable to identify the antigen in this study, because results for the Staphylococcal enterotoxin and SARS-CoV-2 antigen in the intestinal fluid were negative. Nonetheless, further research is needed to clarify the association between the functional role of the SARS-CoV-2 spike protein as a superantigen and ulcerative colitis.

The patient developed ulcerative colitis symptoms one month after the SARS-CoV-2 infection and these symptoms were exacerbated by the first vaccination with BNT162b2. The anti-SARS-CoV-2 spike IgG antibody titer at diagnosis, 13 days after the first vaccination with BNT162b2, was markedly high and decreased accordingly as she recovered from clinical symptoms. Cross-reactivity between SARS-CoV-2 spike proteins or anti-SARS-CoV-2 spike antibodies and human tissues, including the gastrointestinal tract, can increase autoimmune disease (26, 27). The results of the current case support this hypothesis. The antibody titer was measured at month 2.5, which was 1 day after the second BNT162b2 vaccination. It was reported that the antibody titer reached the maximum level 5 to 8 days after the first vaccination and did not significantly exceed the level after the second vaccination (28). Therefore, it might be reasonable that the clinical course of our patients was not apparently influenced by the second vaccination. Further research is needed to determine the functional role of the anti-SARS-CoV-2 antibody as an enhancer of ulcerative colitis or as an autoantibody, in addition to that of the SARS-CoV-2 spike protein.

## 5 Conclusion

SARS-CoV-2 infection may trigger the *de novo* occurrence of ulcerative colitis by impairing barrier function in the intestinal mucosa, T cell activation with a skewed T cell receptor repertoire, and increasing anti-SARS-CoV-2 spike IgG antibody levels. Further research is needed to clarify the association between the functional role of the SARS-CoV-2 spike protein as a superantigen and ulcerative colitis.

## Data availability statement

The original contributions presented in the study are included in the article/**Supplementary Material**. Further inquiries can be directed to the corresponding author.

## Ethics statement

The studies involving human participants were reviewed and approved by University of Tsukuba, Clinical Research Ethics Review Committee. Written informed consent to participate in this study was provided by the participants’ legal guardian/next of kin. Written informed consent was obtained from the individual and minor’s legal guardian/next of kin for the publication of any potentially identifiable images or data included in this article.

## Author contributions

AM and KI conceptualized and designed the study, collected the experimental data, drafted the initial manuscript, and reviewed and revised the manuscript. MT collected the clinical data, carried out the initial analyses, and reviewed and revised the manuscript. NS evaluated histopathology, and reviewed and revised the manuscript. HT coordinated and supervised data collection, and critically reviewed the manuscript. All authors contributed to the article and approved the submitted version.
