# Supplementary material for: Case report: Immunological characteristics of de novo ulcerative colitis in a child post COVID-19
Source: Front Immunol. 2023 Feb 16;14:1107808. doi: 10.3389/fimmu.2023.1107808 (PMC9978098; doi:10.3389/fimmu.2023.1107808)

Supplementary table 1. Panel design for peripheral T-cell subsets

Fluorochrome	Antigen	Clone	Company
		Panel A	
PE-Cy7	CD56	HCD56	Biolegend
eFlour 450	CD19	HIB19	ThermoFisher
eFlour 506	CD3	UCHT1	ThermoFisher
Super Bright 600	CD45	2D1	ThermoFisher
		Panel B	
FITC	TCRγδ	11F2	Miltenyi Biotec
PE	CD38	HIT2	Biolegend
PerCP-eFlour 710	CCR7	3D12	ThermoFisher
PE-Cy7	HLA-DR	L243	ThermoFisher
APC	TCRαβ	IP26	Biolegend
APC-Cy7	CD8	HIT8a	Biolegend
eFlour 450	CD4	RPA-T4	ThermoFisher
eFlour 506	CD3	UCHT1	ThermoFisher
Super Bright 600	CD45RA	HI100	ThermoFisher
		Panel C	
Alexa Flour 488	CD25	M-A251	Biolegend
PE	α4β7	Hu117	R&D systems
PerCP-Cy5.5	CCR6	G034E3	Biolegend
PE-Cy7	CD45RO	UCHL1	ThermoFisher
APC	CXCR5	MU5UBEE	ThermoFisher
APC-Cy7	CXCR3	G025H7	Biolegend
eFlour 450	CD4	RPA-T4	ThermoFisher
eFlour 506	CD3	UCHT1	ThermoFisher
Super Bright 600	CD45RA	HI100	ThermoFisher
Super Bright 702	CD127	eBioRDR5	ThermoFisher

Supplementary table 2. Panel design for T-cell receptor repertories

Fluorochrome	Antigen	Clone	Company
FITC<br>PE	TCR set (A ~ H)		Beckman Coulter
PE-Cy7	HLA-DR	L243	ThermoFisher
PerCP-Cy5.5	CD4	SK3	Biolegend
APC	CD45RO	UCHL1	BD
APC-Cy7	CD8	HIT8a	Biolegend
eFlour 506	CD3	UCHT1	ThermoFisher
Super Bright 702	CD38	HB7	ThermoFisher

Supplementary figure 1. Gating strategy for Panel A.

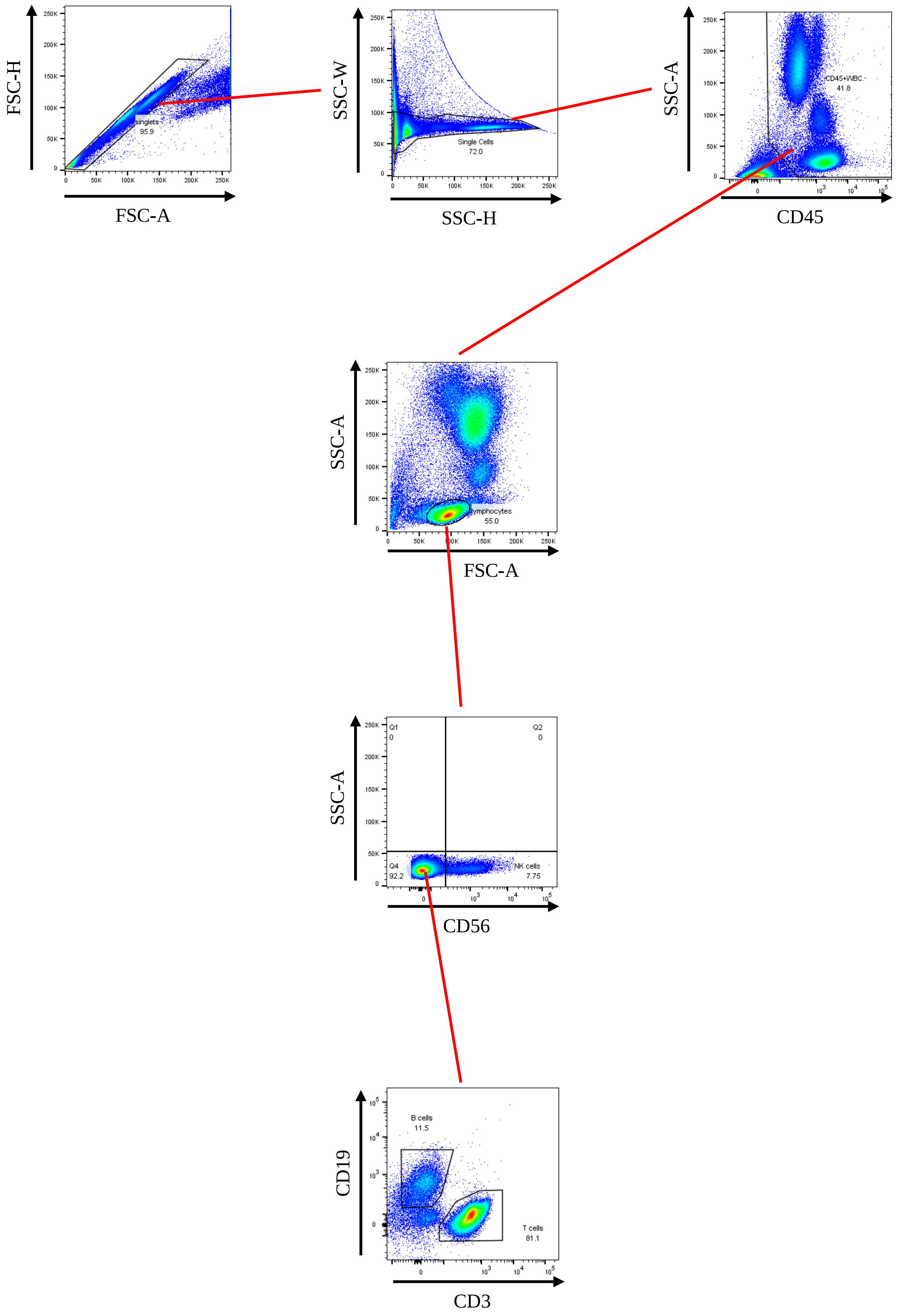

Supplementary figure 2. Gating strategy for Panel B.

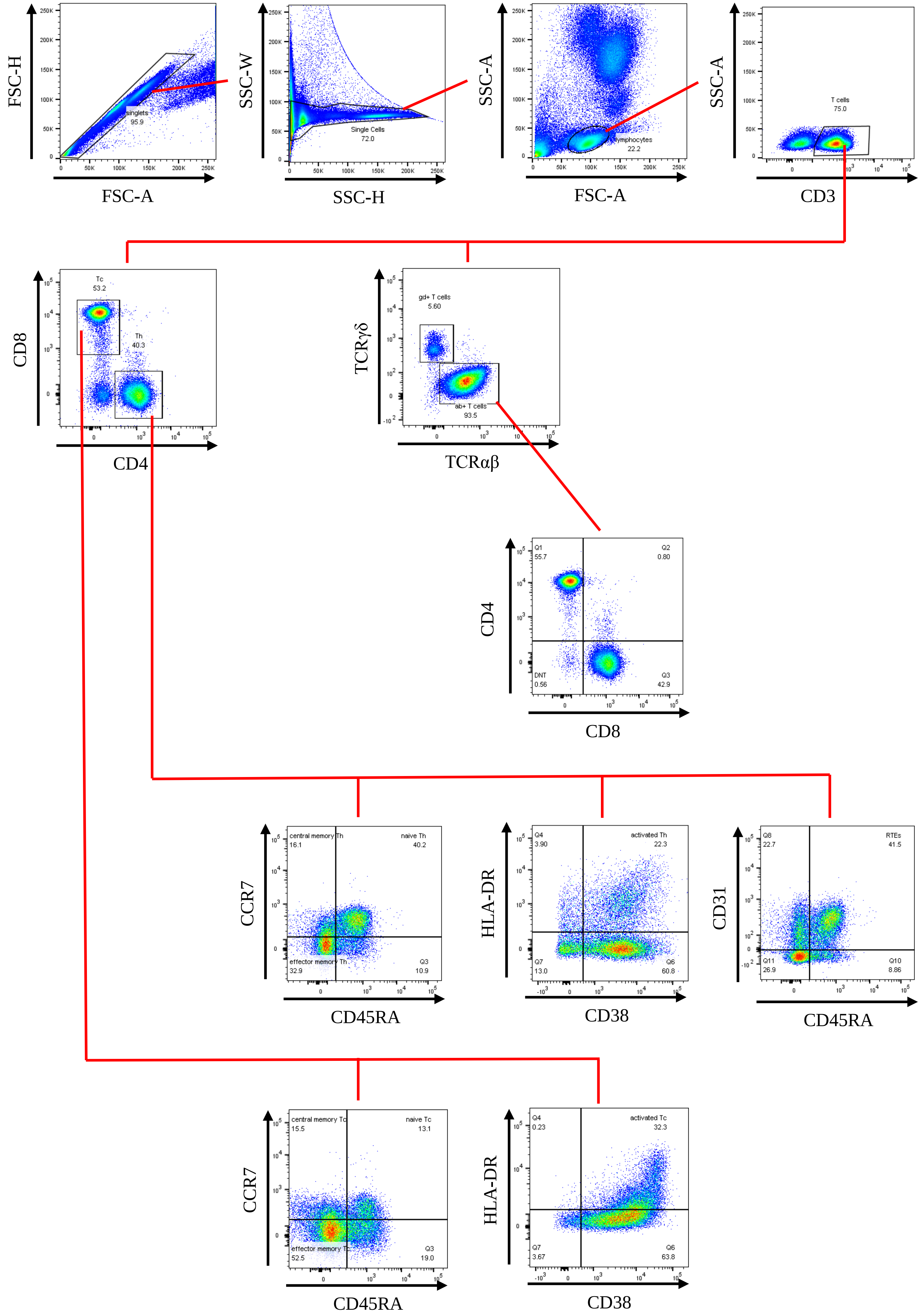

Supplementary figure 3. Gating strategy for Panel C.

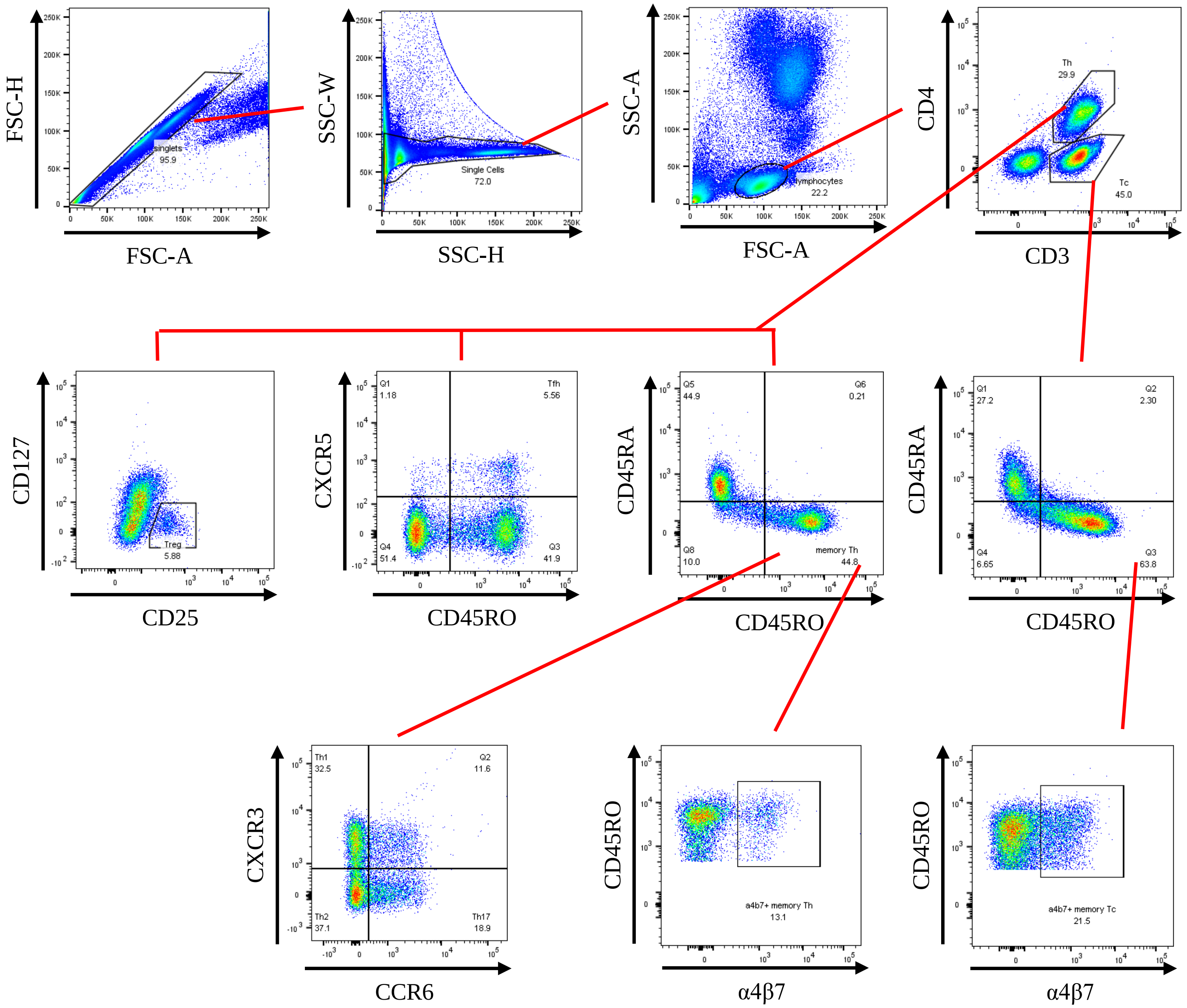

Supplement: Supplementary file 1 [file DataSheet_1.pdf]
